# Diabetes with Hypertension as Risk Factors for Adult Dengue Hemorrhagic Fever in a Predominantly Dengue Serotype 2 Epidemic: A Case Control Study

**DOI:** 10.1371/journal.pntd.0001641

**Published:** 2012-05-01

**Authors:** Junxiong Pang, Agus Salim, Vernon J. Lee, Martin L. Hibberd, Kee Seng Chia, Yee Sin Leo, David C. Lye

**Affiliations:** 1 Infectious Diseases, Genome Institute of Singapore, Singapore, Singapore; 2 Saw Swee Hock School of Public Health, National University of Singapore, Singapore, Singapore; 3 Department of Clinical Epidemiology, Tan Tock Seng Hospital, Singapore, Singapore; 4 Department of Infectious Diseases, Tan Tock Seng Hospital, Singapore, Singapore; 5 Department of Medicine, Yong Loo Lin School of Medicine, National University of Singapore, Singapore, Singapore; Pediatric Dengue Vaccine Initiative, United States of America

## Abstract

**Background:**

Dengue hemorrhagic fever (DHF) is a severe form of dengue, characterized by bleeding and plasma leakage. A number of DHF risk factors had been suggested. However, these risk factors may not be generalized to all populations and epidemics for screening and clinical management of patients at risk of developing DHF. This study explored demographic and comorbidity risk factors for DHF in adult dengue epidemics in Singapore in year 2006 (predominantly serotype 1) and in year 2007–2008 (predominantly serotype 2).

**Methods:**

A retrospective case-control study was conducted with 149 DHF and 326 dengue fever (DF) patients from year 2006, and 669 DHF and 1,141 DF patients from year 2007–2008. Demographic and reported comorbidity data were collected from patients previously. We performed multivariate logistic regression to assess the association between DHF and demographic and co-morbidities for year 2006 and year 2007–2008, respectively.

**Results:**

Only Chinese (adjusted odds ratio [AOR] = 1.90; 95% confidence interval [CI]: 1.01–3.56) was independently associated with DHF in year 2006. In contrast, age groups of 30–39 years (AOR = 1.41; 95% CI:1.09–1.81), 40–49 years (AOR = 1.34; 95% CI:1.09–1.81), female (AOR = 1.57; 95% CI:1.28–1.94), Chinese (AOR = 1.67; 95% CI:1.24–2.24), diabetes (AOR = 1.78; 95% CI:1.06–2.97), and diabetes with hypertension (AOR = 2.16; 95%CI:1.18–3.96) were independently associated with DHF in year 2007–2008. Hypertension was proposed to have effect modification on the risk of DHF outcome in dengue patients with diabetes. Chinese who had diabetes with hypertension had 2.1 (95% CI:1.07–4.12) times higher risk of DHF compared with Chinese who had no diabetes and no hypertension.

**Conclusions:**

Adult dengue patients in Singapore who were 30–49 years, Chinese, female, had diabetes or diabetes with hypertension were at greater risk of developing DHF during epidemic of predominantly serotype 2. These risk factors can be used to guide triaging of patients who require closer clinical monitoring and early hospitalization in Singapore, when confirmed in more studies.

## Author Summary

Dengue is a major vector borne disease in the tropical and subtropical regions. An estimated 50 million infections occur per annum in over 100 countries. A severe form of dengue, characterized by bleeding and plasma leakage, known as dengue hemorrhagic fever (DHF) is estimated to occur in 1–5% of hospitalized cases. It can be fatal if unrecognized and not treated in a timely manner. Previous studies had found a number of risk factors for DHF. However, screening and clinical management strategies based on these risk factors may not be applicable to all populations and epidemics of different serotypes. In this study, we found significant association between DHF and diabetes mellitus and diabetes mellitus with hypertension during the epidemic of predominantly serotype 2 (year 2007 and 2008), but not during the epidemic of predominantly serotype 1 (year 2006). Diabetes mellitus and hypertension are prevalent in Singapore and most parts of South-East Asia, where dengue is endemic. Therefore, it is important to address the risk effect of these co-morbidities on the development of DHF so as to reduce morbidity and mortality. Our findings may have impact on screening and clinical management of dengue patients, when confirmed in more studies.

## Introduction

Dengue is a major neglected tropical disease in the tropical and subtropical regions of the world [1]. It is predominantly found in urban and semi-urban areas, and results in a wide spectrum of clinical manifestations, from asymptomatic infection, undifferentiated fever, dengue fever (DF) to severe infection known as dengue hemorrhagic fever (DHF) and dengue shock syndrome (DSS) [2]. It is estimated about 50 million infections occur annually, with 500,000 DHF cases and 22,000 deaths [3]. In several Asian countries, dengue is one of the leading causes of hospitalization and death among children [1]–[3]. In Singapore, however, there is a decreasing trend of children (aged <15 years) and an increasing trend of adults (aged ≥25 years) being infected with dengue since 1982 [4].

Dengue hemorrhagic fever is characterized by bleeding and plasma leakage which may lead to life-threatening shock, if unrecognized and not treated in a timely manner. Molecular determinants of DHF such as virus variation, viral load, antibody-dependent enhancement (ADE), ‘original antigenic sin’, ‘cytokine storm’ and plasma factors were proposed in the pathophysiology of DHF [5]–[7]. However, predicting or preventing the occurrence of DHF remains a challenge. Identifying risk factors for DHF can facilitate early clinical, preventive and healthcare resource management. Epidemiological risk factors of DHF such as dengue-serotype 2 [8], [9], Asian genotype [10], prior dengue infections [11], [12], children [13], [14], age >65 years [15], white females [12], [16] were identified. Integrative analysis of these risk factors, together with the molecular determinants of DHF, may facilitate better understanding of the pathophysiology of DHF.

Co-morbidities were reported as risk factors for DHF in a number of studies from dengue endemic countries. These co-morbidities included sickle cell anemia [17], asthma [17]–[19], hypertension [15], [16], [18], uremia [15], allergies treated with corticosteroid [16] and diabetes mellitus [15]–[18]. However, these co-morbidities may not be generalized to all populations and epidemics of all dengue serotypes. Furthermore, most of these risk factors were identified from univariate analysis [15], [17]–[19] instead of multivariate analysis to adjust for potential confounders [16]. In this study, we explored demographic and co-morbidity risk factors for DHF in Singapore in the year 2006 (where dengue serotype 1 predominated) and in the year 2007 and 2008 (where dengue serotype 2 predominated) [20].

## Methods

A retrospective case-control study was conducted using data collected from all adult dengue patients admitted from 1 January 2006 to 31 December 2008 to the Department of Infectious Diseases at Tan Tock Seng Hospital (TTSH). It is the largest hospital in Singapore for the treatment of dengue patients where dengue patients were managed using a standardized dengue care path. The extracted data was de-identified in analysis. Dengue fever was defined as reported fever or measured temperature of >38°C with any two of the following manifestations (according to WHO 1997 criteria [2]): headache, retro-orbital pain, myalgia, arthralgia, rash, hemorrhagic manifestations, or leukopenia. Probable dengue patients had positive acute dengue serology, as measured by Dengue Duo IgM & IgG Rapid Strip Test (Panbio Diagnostic, Queensland, Australia) [21], [22], and clinical diagnostic criteria of dengue fever by WHO 1997 [2]. The IgG test line for this Rapid Strip Test is set to detect the high levels of IgG characteristic of secondary virus infection, and has been validated to detect acute secondary infection [22]. Confirmed dengue patients had positive dengue polymerase chain reaction (PCR) assay [23] and clinical diagnostic criteria according to WHO 1997 [2]. Dengue hemorrhagic fever was diagnosed when all four criteria of fever, hemorrhagic manifestations, thrombocytopenia (<100×10^9^/L) and evidence of plasma leakage (hematocrit change ≥20%, hypoproteinemia or clinical fluid accumulation) were present [2].

Patients with outcome diagnosed as DHF and DF were classified as the case group and control group respectively. In order to compare the demographic and co-morbidity profiles between DHF and DF patients, data were extracted from chart review. Demographic data extracted were age, gender and ethnicity. Co-morbidities data extracted were diabetes mellitus, hypertension, asthma, hyperlipidemia, stroke, chronic obstructive pulmonary disease, corticosteroid use and HIV/AIDS. However, stroke, chronic obstructive pulmonary disease, corticosteroid use and HIV/AIDS were not included in the analysis because these co-morbidities were either not reported or reported by very few cases (DHF) or controls (DF). Patients with diabetes mellitus tend to have other co-morbidities, and the risk effect of diabetes mellitus with an additional co-morbidity on DHF outcome was also investigated.

### Statistical methods

For descriptive analysis, Pearson's chi-square and Fisher's exact tests were used to compare categorical variables, and Mann-Whitney U test was used to compare continuous variables with non-normal distribution. Univariate and multivariate logistic regression were used to calculate crude and adjusted odds ratios (COR, AOR), respectively, and their 95% confidence intervals (CI) were used to assess the association of the variables with DHF. Confounding effect was minimized by performing multivariate logistic regression adjusting for potential confounders identified in the descriptive analysis. These potential confounders are exposures that were found to be statistically different (p<0.05) between DHF and DF patients in Table 1 and the model that fits best in the multivariate regression is tested using likelihood-ratio test. In Singapore, dengue infections were predominantly due to dengue serotype 1 (detected in 75% to 100% of dengue samples collected each month) during the epidemic in the year 2006, and dengue serotype 2 (detected in up to 91% of dengue samples) during the epidemic in the year 2007 and 2008 [20]. Given that different dengue serotypes may cause different disease severity [24], the data from year 2006 and the data from year 2007 to 2008 were analysed separately to minimize confounding effect due to different predominant dengue serotypes. Stratified analyses were performed to evaluate the presence of effect modification between diabetes mellitus and other co-morbidities on the risk of DHF outcome. All statistical analyses were performed using Stata 10.0 (Stata Corp., College Station, TX, 2005). All tests were conducted at the 5% level of significance, with OR, P-value and corresponding 95% CI reported where applicable.

**Table 1:** Distribution of demographic characteristics and co-morbidities by cases (DHF) and controls (DF) in year 2006 and year 2007–2008 epidemics.

Year	2006 (N = 475)						2007–2008 (N = 1810)					
Exposure	Cases (N = 149)		Controls (N = 326)		Case ∶ Control		Cases (N = 669)		Controls (N = 1141)		Case ∶ Control	
	N	%	N	%		P-valueΔ	N	%	N	%		P-valueΔ
**Age (Years)**												
Mean (SD)	37.3	(12.8)	34.0	(11.0)		**0.008** ∧	38.4	(13.4)	36.2	(12.9)		**<0.001** ∧
<30	43	28.9	115	35.3	1 ∶ 2.67		180	26.9	405	35.5	1 ∶ 2.25	
30–39	46	30.9	127	39.0	1 ∶ 2.76		209	31.2	328	28.8	1 ∶ 1.57	
40–49	39	26.2	58	17.8	1 ∶ 1.49		152	22.7	213	18.7	1 ∶ 1.40	
50–59	12	8.1	19	5.8	1 ∶ 1.58		76	11.4	132	11.6	1 ∶ 1.74	
≥60	9	6.0	7	2.2	1 ∶ 0.78	**0.017**	52	7.8	63	5.5	1 ∶ 1.21	**0.002**
**Gender**												
Male	101	67.8	233	71.5	1 ∶ 2.31		393	58.7	809	70.9	1 ∶ 2.06	
Female	48	32.2	93	28.5	1 ∶ 1.94	0.414	276	41.3	332	29.1	1 ∶ 1.20	**<0.001**
**Ethnicity**												
Others	15	10.1	60	18.4	1 ∶ 4.00		79	11.8	188	16.5	1 ∶ 2.38	
Chinese	115	77.2	205	62.9	1 ∶ 1.78		516	77.1	717	62.8	1 ∶ 1.39	
Malay	4	2.7	12	3.7	1 ∶ 3.00		38	5.7	62	5.4	1 ∶ 1.63	
Indian	15	10.1	49	15.0	1 ∶ 3.27	**0.021**	36	5.4	174	15.3	1 ∶ 4.83	**<0.001**
**Fever DBP**												
Mean (SD)	4.9	(1.5)	4.9	(1.6)		0.941∧	4.9	(1.7)	5.0	(1.8)		0.308∧
**Hypertension**												
No	140	94.0	315	96.6	1 ∶ 2.25		594	88.8	1047	91.8	1 ∶ 1.76	
Yes	9	6.0	11	3.4	1 ∶ 1.22	0.179	75	11.2	94	8.2	1 ∶ 1.25	**0.036**
**Diabetes**												
No	147	98.7	319	97.9	1 ∶ 2.17		626	93.6	1101	96.5	1 ∶ 1.76	
Yes	2	1.3	7	2.2	1 ∶ 3.50	0.726#	43	6.4	40	3.5	1 ∶ 0.93	**0.004**
**Hyperlipidemia**												
No	145	97.3	317	97.2	1 ∶ 2.19		612	91.5	1061	93.0	1 ∶ 1.73	
Yes	4	2.7	9	2.8	1 ∶ 2.25	1.000#	57	8.5	80	7.0	1 ∶ 1.40	0.241
**Asthma**												
No	145	97.3	311	95.4	1 ∶ 2.15		637	95.2	1082	94.8	1 ∶ 1.70	
Yes	4	2.7	15	4.6	1 ∶ 3.75	0.323	32	4.8	59	5.2	1 ∶ 1.84	0.740

**Δ:** Person's Chi-square, unless otherwise annotated.

**∧:** Mann-Whitney U test.

# Fisher's Exact test.

DHF-Dengue hemorrhagic fever.

DF- Dengue fever.

DBP- Days before presentation in hospital.

### Ethics approval

This study was approved by Domain Specific Review Board, National Healthcare Group, Singapore (DSRB-E/08/567) with waiver of informed consent as this was a retrospective study and the data were analyzed anonymously.

## Results

### Demographic and co-morbidity profiles of DHF & DF patients during the two epidemics

In year 2006 epidemic, there were 149 DHF and 326 DF patients. Among these patients, there were 131 (27.6%) patients who were PCR positive and 344 (72.4%) patients who were serology positive but PCR negative. The mean age was 37.3 (±12.8) years and 34.0 (±11.0) years for DHF patients and DF patients respectively. Among DHF patients, there were 67.8% male and 77.2% Chinese. Of the 326 DF patients, 71.5% were male and 62.9% were Chinese (Table 1). In year 2007 and 2008 epidemic, there were 669 DHF and 1,141 DF patients. Among these patients, there were 590 (32.6%) patients who were PCR positive and 1220 (67.4%) patients who were serology positive but PCR negative. The mean age was 38.4 (±13.4) years and 36.2 (±12.9) years for DHF patients and DF patients respectively. Among the DHF patients, there were 58.7% male and 77.1% Chinese. Of the 1,141 DF patients, 70.9% were male and 62.8% were Chinese (Table 1).

Of the demographic variables, statistically significant differences (P<0.05) were found between DHF and DF with respect to mean age (P = 0.008), age groups (P = 0.017) and ethnicity (P = 0.021) in year 2006, and mean age (P<0.001), age groups (P = 0.002), gender (P<0.001) and ethnicity (P<0.001) in year 2007 and 2008 (Table 1). Using the number of fever days before hospital presentation as a surrogate index of health-seeking behavior between DHF and DF patients, no significant difference was observed in both year 2006 (P = 0.941) as well as year 2007–2008 (P = 0.308) (Table 1). Notably, statistically significant differences were found between DHF and DF with respect to hypertension (P = 0.036) and diabetes mellitus (P = 0.004) in year 2007 and 2008 but not year 2006 (Table 1).

### Independent risk factors for DHF

Chinese ethnicity was the only significant risk factor independently associated with DHF in year 2006, after adjustment for statistically significant univariate risk factors (Table 2). Although marginally significant, the likelihood (AOR) of a Chinese patient developing DHF was 1.90 (95% CI:1.01–3.56) times higher than that of other ethnicity (not Chinese, Malay or Indian). In year 2007 and 2008, age groups, gender and ethnicity were observed to be independently associated with DHF, following adjustment for statistically significant univariate risk factors (Table 2). The likelihood (AOR) of an individual who were 30 to 39 years of age and 40 to 49 years of age developing DHF was 1.41 (95% CI:1.09–1.81) and 1.34 (95% CI:1.09–1.81) times higher than that of an individual below 30 years old respectively. Females had 1.57 (95% CI:1.28–1.94) times higher risk developing DHF than males. In addition, the likelihood (AOR) of a Chinese patient developing DHF was 3.15 (95% CI:2.34–4.23) and 1.67 (95% CI:1.24–2.24) times higher than that of Indian and other ethnicity respectively (Table 2).

**Table 2:** Crude and adjusted odds ratios of the association of DHF with age groups, gender and ethnicity in year 2006 and year 2007–2008 epidemics.

Year	2006				2007–2008			
Exposure	COR	95% CI	AOR*	95% CI	COR	95% CI	AOR**	95% CI
**Age (Years)**								
<30	1		1		1		1	
30–39	0.97	0.60–1.58	0.92	0.56–1.50	**1.43**	**1.12–1.84**	**1.41**	**1.09–1.81**
40–49	**1.80**	**1.05–3.07**	1.53	0.88–2.67	**1.61**	**1.22–2.11**	**1.34**	**1.09–1.81**
50–59	1.69	0.76–3.77	1.47	0.65–3.31	1.30	0.93–1.81	0.91	0.63–1.30
≥60	**3.44**	**1.20–9.81**	2.71	0.94–7.88	**1.86**	**1.24–2.79**	1.14	0.70–1.85
**Gender**								
Male	1		1		1		1	
Female	1.19	0.78–1.81	1.14	0.74–1.78	**1.71**	**1.40–2.09**	**1.57**	**1.28–1.94**
**Ethnicity**								
Others	1		1		1		1	
Chinese	**2.24**	**1.22–4.13**	**1.90**	**1.01–3.56**	**1.71**	**1.29–2.28**	**1.67**	**1.24–2.24**
Malay	1.33	0.38–4.72	1.15	0.32–4.13	1.46	0.90–2.36	1.30	0.79–2.13
Indian	1.22	0.55–2.75	1.19	0.53–2.68	**0.49**	**0.32–0.77**	**0.53**	**0.34–0.83**

*: Adjusted odds ratio was obtained from a multivariate logistic regression being adjusted by age groups and ethnicity.

**: Adjusted odds ratio was obtained from a multivariate logistic regression being adjusted by age groups, gender, ethnicity, diabetes mellitus and hypertension.

DHF- Dengue Hemorrhagic Fever.

COR- Crude odds ratio.

AOR- Adjusted odds ratio.

CI- Confidence interval.

For co-morbidities, after adjustment for statistically significant univariate risk factors, only diabetes mellitus remained an independent risk factor for DHF outcome (AOR = 1.78; 95% CI:1.06–2.97) in year 2007 and 2008 (Table 3). Diabetic patients tend to have other co-morbidities. We investigated the risk effect of DHF outcome on patients having diabetes mellitus with hypertension, hyperlipidemia or asthma. Diabetes mellitus with hypertension (COR = 2.43; 95% CI:1.42–4.15), diabetes mellitus with hyperlipidemia (COR = 1.82; 95% CI:1.06–3.12) and diabetes mellitus with no asthma (COR = 1.74; 95% CI:1.10–2.76) were observed to be significantly associated with DHF outcome (Table 4). However, only diabetes mellitus with hypertension (AOR = 2.16; 95% CI:1.18–3.96) and diabetes with no asthma (AOR = 1.68; 95% CI:1.02–2.76) were observed to be independently associated with DHF outcome after adjustment for statistically significant univariate risk factors (Table 4). Interestingly, the likelihood (AOR) of an individual having diabetes mellitus with asthma developing DHF was 4.38 (95% CI:0.80–23.85) times higher than that of an individual having no diabetes with no asthma. However, there is a lack of statistical significance and it is most likely due to the small sample size with only 7 subjects having diabetes mellitus with asthma (Table 4). In order to confirm this observed phenomenon, further studies with larger sample size are required. In addition, among patients with hypertension, the likelihood (AOR) of developing DHF due to diabetes mellitus was higher (AOR = 2.39; 95% CI:1.21–4.71) compared to that of patients without hypertension (AOR = 1.28; 95% CI:0.56–2.93; Table 5). This provided preliminary evidence of effect modification between diabetes mellitus and hypertension on the risk of DHF outcome. Moreover, it was observed that the mean hospitalization days was longer for diabetic patients (4.99±3.34 days) compared to non-diabetic patients (4.04±1.62 days; P = 0.001). Significant difference was also observed in the mean hospitalization days between diabetic DHF patients and non-diabetic DHF patients (diabetic DHF: 5.21±3.12 days; non-diabetic DHF: 4.33±1.75 days; P = 0.046) (data not shown).

**Table 3:** Crude and adjusted odds ratios of the association of DHF with co-morbidities in year 2006 and year 2007–2008 epidemics.

Year	2006				2007–2008			
Exposure	COR	95% CI	AOR*	95% CI	COR	95% CI	AOR**	95% CI
**Hypertension**								
No	1		1		1		1	
Yes	1.84	0.74–4.54	0.97	0.31–3.00	**1.41**	**1.02–1.94**	1.06	0.70–1.60
**Diabetes**								
No	1		1		1		1	
Yes	0.62	0.13–3.02	0.34	0.06–1.89	**1.89**	**1.21–2.94**	**1.78**	**1.06–2.97**
**Hyperlipidemia**								
No	1		1		1		1	
Yes	0.97	0.29–3.20	0.54	0.15–1.96	1.24	0.87–1.76	0.79	0.50–1.26
**Asthma**								
No	1		1		1		1	
Yes	0.57	0.19–1.75	0.51	0.16–1.62	0.92	0.59–1.43	0.86	0.55–1.35

*: Adjusted Odds Ratio was obtained from a multivariate logistic regression being adjusted by age groups and ethnicity.

**: Adjusted odds ratio was obtained from a multivariate logistic regression being adjusted by age groups, gender, ethnicity, diabetes mellitus and hypertension.

DHF- Dengue Hemorrhagic Fever.

COR- Crude odds ratio.

AOR- Adjusted odds ratio.

CI- Confidence interval.

**Table 4:** Crude and adjusted odds ratios of the association of DHF with multiple co-morbidities in year 2007–2008 epidemic.

Exposures	Cases	Controls				
	N	N	COR	95% CI	AOR*	95% CI
**Diabetes**						
No	626	1101	1		1	
Yes	43	40	**1.89**	**1.21–2.94**	**1.78**	**1.06–2.97**
**Diabetes, Hypertension**						
No diabetes with no hypertension	584	1031	1		1	
No diabetes with hypertension	42	70	1.06	0.71–1.57	0.97	0.62–1.52
Diabetes with no hypertension	10	16	1.1	0.50–2.45	1.26	0.55–2.87
Diabetes with hypertension	33	24	**2.43**	**1.42–4.15**	**2.16**	**1.18–3.96**
**Diabetes, Hyperlipidemia**						
No diabetes with no hyperlipidemia	597	1048	1		1	
No diabetes with hyperlipidemia	29	53	0.96	0.60–1.53	0.82	0.50–1.37
Diabetes with no hyperlipidemia	15	13	2.03	0.96–4.29	2.03	0.93–4.47
Diabetes with hyperlipidemia	28	27	**1.82**	**1.06–3.12**	1.62	0.90–2.92
**Diabetes, Asthma**						
No diabetes with no asthma	599	1044	1		1	
No diabetes with asthma	27	57	0.83	0.52–1.32	0.79	0.49–1.27
Diabetes with no asthma	38	38	**1.74**	**1.10–2.76**	**1.68**	**1.02–2.76**
Diabetes with asthma	5	2	4.36	0.84–22.53	4.38	0.80–23.85

*: Adjusted odds ratio was obtained from a multivariate logistic regression being adjusted by age groups, gender, ethnicity, diabetes mellitus and hypertension.

DHF- Dengue Hemorrhagic Fever.

COR- Crude odds ratio.

AOR- Adjusted odds ratio.

CI- Confidence interval.

**Table 5:** Interaction effect between diabetes mellitus and other comorbidities in year 2007–2008 epidemic.

Exposures	Cases	Controls				
	N	N	COR	95% CI	AOR*	95% CI
**Diabetes**						
No	626	1101	1		1	
Yes	43	40	**1.89**	**1.21–2.94**	**1.78**	**1.06–2.97**
**No hypertension**						
No diabetes	584	1031	1		1	
Diabetes	10	16	1.10	0.50–2.45	1.28	0.56–2.93
**Hypertension**						
No diabetes	42	70	1		1	
Diabetes	33	24	**2.29**	**1.20–4.39**	**2.39**	**1.21–4.71**
**No hyperlipidemia**						
No diabetes	597	1048	1		1	
Diabetes	15	13	2.03	0.96–4.29	1.95	0.88–4.36
**Hyperlipidemia**						
No diabetes	29	53	1		1	
Diabetes	28	27	1.90	0.94–3.80	2.03	0.93–4.41
**No asthma**						
No diabetes	599	1044	1		1	
Diabetes	38	38	**1.74**	**1.10–2.76**	**1.77**	**1.03–3.00**
**Asthma**						
No diabetes	27	57	1		1	
Diabetes	5	2	5.28	0.96–29.0	1.01	0.04–25.15

*: Adjusted odds ratio was obtained from a multivariate logistic regression being adjusted by age groups, gender, ethnicity, diabetes mellitus and hypertension.

DHF- Dengue Hemorrhagic Fever.

COR- Crude odds ratio.

AOR- Adjusted odds ratio.

CI- Confidence interval.

Subgroup analyses of patients with dengue IgG data and of Chinese patients were carried out. In the subgroup analysis of 1,220 (67.4%) patients hospitalized during the year 2007–2008 that had dengue IgG data, we further showed that diabetes (AOR: 1.92; 95% CI: 1.02–3.61) as well as diabetes with hypertension (AOR: 4.41; 95% CI: 1.16–16.82) remained as risk factor of DHF (Table S1). Furthermore, in a subgroup analysis of cases (DHF) and controls (DF) identified as Chinese in year 2007 and 2008, diabetes mellitus (AOR = 2.23; 95% CI:1.21–4.11), diabetes mellitus with hypertension (AOR = 2.1; 95% CI:1.07–4.12), diabetes mellitus with no hyperlipidemia (AOR = 3.75; 95% CI:1.27–11.02) and diabetes mellitus with no asthma (AOR = 1.96; 95% CI:1.09–3.52) were independently associated with DHF outcome, after adjustment for age groups, gender, and hypertension (data not shown).

## Discussion

The results of this study showed that female, Chinese, age group between 30 to 49 years, pre-existing diabetes mellitus or diabetes mellitus with hypertension were risk factors of developing DHF during the year 2007 and 2008 epidemic when dengue serotype 2 was predominant. In contrast, Chinese ethnicity was the only risk factor observed during the year 2006 epidemic when dengue serotype 1 was predominant. This might be due to the different predominant circulating dengue serotypes during the two epidemics [20]. Notably, dengue serotype 2 was known to be associated with more severe dengue disease than serotype 1 [8], [9], [24]. In a combined analysis of year 2006, 2007 and 2008 epidemic, all the risk factors identified in the year 2007–2008 epidemic remained as independent risk factors except for diabetes mellitus (Table S2). This may suggest potential confounding effect of different serotypes. Furthermore, it was observed that age, gender and co-morbidities were not independently associated with DHF outcome in a previous study of 1,973 adult dengue patients in the year 2004 epidemic when dengue serotype 1 was also predominant [25]. However, it is not possible to conclusively demonstrate serotype difference during epidemics as the main factor that accounted for the differences in risk factors in this study. This was because individual serotype data was inaccessible for our analyses due to national regulations. Instead, the differences in risk factors may be due to the small sample size of patients admitted in year 2006, and the significant differences in mean age, number of patients with co-morbidities and DHF outcome admitted during the two epidemics (Table S3). It is beyond the scope of this study to highlight other potential factors, such as climate change, viral genotype change as well as change in health-seeking behaviors that may have also resulted in the differences.

It is not surprising that female and Chinese ethnicity were independent risk factors of DHF as gender [12], [26] and ethnicity [12], [16] were shown to be risk factors for DHF in Cuba and Brazil studies as well as in Vietnam for dengue shock syndrome (DSS). Age groups between 30 and 39 and between 40 and 49 were independent risk factors of DHF in our adult dengue cohort. This observation is different from previous studies in Cuba [13] and in Singapore [14] where children <14 years of age had higher risk of developing DHF compared to young adults aged 15 years or greater. The rationale behind this difference could be due to lowered herd immunity and change in transmission pattern [14], [27]. The elderly (>65 years of age) in Taiwan [15] had a higher risk of developing DHF. However, the age group ≥60 year was not an independent risk factor of DHF outcome (Table 2) in our current study which is also consistent with our previous study on dengue in older adults [28]. It is still not well understood how these risk factors contribute to the pathophysiology of DHF, and understanding the underlying mechanism may facilitate clinical management.

Co-morbidities such as hypertension, diabetes mellitus, hyperlipidemia and asthma are among the few leading causes of mortality and morbidity in Asia [29] and globally [30], [31]. Co-morbidities were shown to be associated with severe clinical manifestations of several infectious disease such as SARS [32], [33], pandemic influenza H1N1 [34], tuberculosis [35], [36], hepatitis C [37] and community-acquired infections [38], [39]. Many studies found association between various co-morbidities and DHF outcome [15]–[19] but only one study was carried out with multivariate analysis to adjust for potential confounders [16]. Furthermore, none has evaluated the risk effect of two existing co-morbidities and the effect modification between two co-morbidities on DHF outcome. In this study, we showed that diabetes mellitus was associated with DHF outcome as observed by other studies [15]–[18]. In addition, we observed that individuals reported having diabetes mellitus with hypertension had higher risk of developing DHF compared with individuals with no diabetes mellitus and no hypertension. Our study may be the first that provide the preliminary evidence of synergy of risk effect between diabetes mellitus and hypertension on DHF outcome (Tables 4 & 5). Our study showed concomitant diabetes mellitus with hypertension as an independent risk factor for DHF in a large number of adult DHF cases in Singapore, and supported the initial evidence of association between hospitalization with a diagnosis of DHF and diabetes mellitus in Brazil [16]. However, the pathophysiology behind diabetes leading to DHF outcome is not well understood yet, even though numerous studies had suggested that diabetes mellitus can result in immune and endothelial dysfunction [40]–[44].

Identifying risk factors for DHF can guide clinicians to triage dengue patients for the right site of care for closer monitoring and early intervention with fluid resuscitation. In an epidemic where healthcare resources may be stretched, risk factors for DHF can be used to prioritize hospitalization of dengue patients. In our study, we observed that diabetic patients with DHF outcome required longer stay and, presumably, required more medical attention in the hospital compared to non-diabetic patients with DHF. Additionally, policy makers can prioritize population groups at high risk of developing DHF such as female patients, patients in age group 30–49, and patients having diabetes or diabetes with hypertension for vaccination when dengue vaccines are available, particularly in resource-limited countries. Demographic and co-morbidity risk factors may help public health clinicians raise awareness among high-risk individuals to take preventive measures against dengue infections.

As this is a retrospective study, the quality of the study was dependent on the quality of the data available and collected. Information bias was minimized by the use of the standardized dengue care path for consistent clinical documentation. Reporting bias was minimized by the fact that patients with comorbidities tend to know their existing condition and are likely to be on constant medication. However, it is challenging to exclude the fact that there are no undetected existing comorbidities among some of these patients as this study is performed retrospectively. In addition, there may be selection bias because the subjects were all hospitalized and hence were likely to have active health care-seeking behaviour, and the controls were hospitalized DF patients who may not truly represent the general population. In the general population, less active health care-seeking, asymptomatic or mild DF patients may not visit a doctor or hospital and may also have diabetes mellitus or other co-morbidities assessed in this study. However, it is technically challenging to identify these less active health care-seeking, asymptomatic or mild DF patients for inclusion in the study. We also did not have patient-specific dengue serotype data and could only extrapolate our observations from previous population study in Singapore [20]. Lastly, we understand the importance of accounting for prior infection as it is a main risk factor for DHF. The result of IgG test carried out within seven days of fever onset can be used to classify patients with or without prior infection [22], [45]. However, we only have had IgG results of 67.4% of all patients during the year 2007–2008. In the subgroup analysis, we showed that prior infection was not significantly associated with DHF in adult patients (Table S1). Furthermore, it has been shown that prior infection was strongly associated with DHF in children under the age of 15 years [24], [46]. In other words, this may suggest that diabetes as well as diabetes with hypertension may be risk factors of DHF in adults, regardless of prior dengue infections. Further studies involving larger number of patients with acute secondary infections are required to confirm this hypothesis.

In conclusion, we found age between 30 and 49 years, female gender, Chinese ethnicity, diabetes mellitus and diabetes mellitus with hypertension to be independent risk factors for DHF in an adult dengue epidemic with predominantly dengue serotype 2. The two co-morbidities appeared to have effect modification on the risk of DHF outcome. More studies, particularly prospective studies are required to confirm these findings. Our finding raised the likely association between the pathophysiology of diabetes mellitus, hypertension and dengue severity. An ongoing genome-wide association study in Singapore may help elucidate genetic predisposition to severe dengue disease including the role of diabetes mellitus.

## Supporting Information

Table S1 **Subgroup analyses of dengue patients with IgG Rapid Test data in year 2007–2008 epidemic.** Subgroup analyses of adult patients with IgG Rapid Test data suggested that female, Chinese, diabetes as well as diabetes with hypertension remain as significant independent risk factors of DHF.
(DOC)

Table S2 **Combined analyses of adult dengue patients admitted in year 2006, 2007 and 2008 epidemic.** Combined analyses of dengue patients admitted in the three consecutive years showed that age group from 30–49, female, Chinese, but not diabetes, are significant independent risk factors for DHF.
(DOC)

Table S3 **Descriptive analysis of demographics and co-morbidities variables between epidemics in year 2006 and year 2007–2008.** There are significant differences in the distribution of age groups, number of adult dengue patients with diabetes, hypertension and hyperlipidemia, as well as the number of DHF cases between the group of patients admitted in year 2006 epidemic and the group admitted in year 2007–2008 epidemic.
(DOC)
