# Supplementary material for: Diabetes with Hypertension as Risk Factors for Adult Dengue Hemorrhagic Fever in a Predominantly Dengue Serotype 2 Epidemic: A Case Control Study
Source: PLoS Negl Trop Dis. 2012 May 1;6(5):e1641. doi: 10.1371/journal.pntd.0001641 (PMC3341340; doi:10.1371/journal.pntd.0001641)
Supplement: Table S1 — Subgroup analyses of dengue patients with IgG Rapid Test data in year 2007–2008 epidemic. Subgroup analyses of adult patients with IgG Rapid Test data suggested that female, Chinese, diabetes as well as diabetes with hypertension remain as significant independent risk factors of DHF. (DOC) [file pntd.0001641.s001.doc]

**Table S1.** Subgroup analyses of patients with IgG Rapid Test data in year 2007-2008 epidemic.

|  | **Case (DHF;N=439)** | | **Control (DF;N=781)** | |  |  |  |  |  |
| --- | --- | --- | --- | --- | --- | --- | --- | --- | --- |
|  | **N** | **%** | **N** | **%** | **P-value^$^** | **COR** | **95% CI** | **AOR*** | **95% CI** |
| **Age (Years)** |  |  |  |  |  |  |  |  |  |
| <30 | 124 | 28.3 | 280 | 35.9 |  | 1 |  | 1 |  |
| 30 – 39 | 134 | 30.5 | 216 | 27.7 |  | **1.4** | **1.04 – 1.89** | 1.33 | 0.97 – 1.82 |
| 40 -49 | 96 | 21.9 | 155 | 19.9 |  | **1.4** | **1.00 – 1.95** | 1.06 | 0.74 – 1.51 |
| 50 – 59 | 55 | 12.5 | 94 | 12.0 |  | 1.32 | 0.89 – 1.96 | 0.88 | 0.57 – 1.35 |
| ≥ 60 | 30 | 6.8 | 36 | 4.6 | 0.064 | **1.88** | **1.11 – 3.19** | 1.08 | 0.60 – 1.94 |
| **Gender** |  |  |  |  |  |  |  |  |  |
| Male | 252 | 57.4 | 532 | 68.1 |  | 1 |  | 1 |  |
| Female | 187 | 42.6 | 249 | 31.9 | **<0.001** | **1.59** | **1.25 – 2.02** | **1.42** | **1.10 – 1.82** |
| **Ethnicity** |  |  |  |  |  |  |  |  |  |
| Others | 57 | 13.0 | 128 | 16.4 |  | 1 |  | 1 |  |
| Chinese | 338 | 77.0 | 483 | 61.8 |  | **1.57** | **1.12 – 2.21** | **1.67** | **1.17 – 2.39** |
| Malay | 25 | 5.7 | 44 | 5.6 |  | 1.28 | 0.71 – 2.28 | 1.23 | 0.68 – 2.23 |
| Indian | 19 | 4.3 | 126 | 16.1 | **<0.001** | **0.34** | **0.19 – 0.60** | **0.35** | **0.20 – 0.63** |
| **IgG** |  |  |  |  |  |  |  |  |  |
| Negative | 152 | 34.6 | 287 | 36.8 |  | 1 |  | 1 |  |
| Positive | 287 | 65.4 | 494 | 63.3 | 0.458 | 1.10 | 0.86 – 1.40 | 1.26 | 0.96 – 1.65 |
| **Hypertension** |  |  |  |  |  |  |  |  |  |
| No | 396 | 90.2 | 722 | 92.5 |  | 1 |  | 1 |  |
| Yes | 43 | 9.8 | 59 | 7.6 | 0.175 | 1.33 | 0.88 – 2.00 | 0.95 | 0.56 – 1.60 |
| **Diabetes** |  |  |  |  |  |  |  |  |  |
| No | 413 | 94.1 | 757 | 96.9 |  | 1 |  | 1 |  |
| Yes | 26 | 5.9 | 24 | 3.1 | **0.016** | **1.98** | **1.13 – 3.50** | **1.92** | **1.02 – 3.61** |
| **Hyperlipidemia** |  |  |  |  |  |  |  |  |  |
| No | 406 | 92.5 | 730 | 93.5 |  | 1 |  | 1 |  |
| Yes | 33 | 7.5 | 51 | 6.5 | 0.513 | 1.16 | 0.74 – 1.83 | 0.76 | 0.44 – 1.33 |
|  |  |  |  |  |  |  |  |  |  |
|  |  |  |  |  |  |  |  |  |  |
|  |  |  |  |  |  |  |  |  |  |
|  | **Case (N=439)** | | **Control (N=781)** | |  |  |  |  |  |
|  | **N** | **%** | **N** | **%** | **P-value**^∆^ | **COR** | **95% CI** | **AOR*** | **95% CI** |
| **Asthma** |  |  |  |  |  |  |  |  |  |
| No | 414 | 94.3 | 747 | 95.7 |  | 1 |  | 1 |  |
| Yes | 25 | 5.7 | 34 | 4.4 | 0.295 | 1.33 | 0.78 – 2.25 | 1.19 | 0.69 – 2.05 |
| **Diabetes, Hypertension** |  |  |  |  |  |  |  |  |  |
| No diabetes with no hypertension | 391 | 89.1 | 708 | 90.7 |  | 1 |  | 1 |  |
| No diabetes with hypertension | 22 | 5.0 | 49 | 6.3 |  | 0.81 | 0.48 – 1.36 | 0.72 | 0.40 – 1.29 |
| Diabetes with no hypertension | 5 | 1.1 | 14 | 1.8 |  | 0.65 | 0.23 – 1.81 | N.A |  |
| Diabetes with hypertension | 21 | 4.8 | 10 | 1.3 | **0.002** | **3.80** | **1.77 – 8.16** | **4.41** | **1.16 – 16.82** |
| **Diabetes, Hyperlipidemia** |  |  |  |  |  |  |  |  |  |
| No diabetes with no hyperlipidemia | 397 | 9.4 | 720 | 92.2 |  | 1 |  | 1 |  |
| No diabetes with hyperlipidemia | 16 | 3.6 | 37 | 4.7 |  | 0.78 | 0.43 – 1.43 | 0.66 | 0.35 – 1.26 |
| Diabetes with no hyperlipidemia | 9 | 2.1 | 10 | 1.3 |  | 1.63 | 0.66 – 4.05 | 0.80 | 0.25 – 2.62 |
| Diabetes with hyperlipidemia | 17 | 3.9 | 14 | 1.8 | 0.081 | **2.20** | **1.07 – 4.51** | N.A |  |
| **Diabetes, Asthma** |  |  |  |  |  |  |  |  |  |
| No diabetes with no asthma | 392 | 89.3 | 725 | 92.8 |  | 1 |  | 1 |  |
| No diabetes with asthma | 21 | 4.8 | 32 | 4.1 |  | 1.21 | 0.69 – 2.13 | 1.14 | 0.64 – 2.02 |
| Diabetes with no asthma | 22 | 5.0 | 22 | 2.8 |  | **1.85** | **1.01 – 3.38** | 0.53 | 0.08 – 3.42 |
| Diabetes with asthma | 4 | 0.9 | 2 | 0.3 | 0.069 | 3.70 | 0.67 – 20.28 | N.A |  |

^∆^ Person’s Chi-square

N.A- Not applicable due to collinearity

*Adjusted odds ratio was obtained from a multivariate logistic regression being adjusted by age groups, gender, ethnicity, IgG status and diabetes mellitus.

DHF-Dengue Hemorrhagic Fever

DF- Dengue Fever

COR- Crude odds ratio

AOR- Adjusted odds ratio

CI- Confidence interval
