# Supplementary material for: Diabetes with Hypertension as Risk Factors for Adult Dengue Hemorrhagic Fever in a Predominantly Dengue Serotype 2 Epidemic: A Case Control Study
Source: PLoS Negl Trop Dis. 2012 May 1;6(5):e1641. doi: 10.1371/journal.pntd.0001641 (PMC3341340; doi:10.1371/journal.pntd.0001641)
Supplement: Table S2 — Combined analyses of adult dengue patients admitted in year 2006, 2007 and 2008 epidemic. Combined analyses of dengue patients admitted in the three consecutive years showed that age group from 30–49, female, Chinese, but not diabetes, are significant independent risk factors for DHF. (DOC) [file pntd.0001641.s002.doc]

**Table S2.** Combined analyses of adult dengue patients admitted in year 2006, 2007 and 2008 epidemic.

|  | **Case (N=818)** | | **Control (N=1467)** | |  |  |  |  |  |
| --- | --- | --- | --- | --- | --- | --- | --- | --- | --- |
|  | **N** | **%** | **N** | **%** | **P-value**^∆^ | **COR** | **95% CI** | **AOR^*^** | **95% CI** |
| **Age (Years)** |  |  |  |  |  |  |  |  |  |
| <30 | 223 | 27.26 | 520 | 35.45 |  | 1 |  | 1 |  |
| 30 - 39 | 255 | 31.17 | 455 | 31.02 |  | 1.31 | **1.05 - 1.63** | 1.29 | **1.03 - 1.61** |
| 40 -49 | 191 | 23.35 | 271 | 18.47 |  | 1.64 | **1.29 - 2.09** | 1.41 | **1.09 -1.81** |
| 50 - 59 | 88 | 10.76 | 151 | 10.29 |  | 1.36 | 1.00 - 1.85 | 1.00 | 0.71 -1.39 |
| ≥ 60 | 61 | 7.46 | 70 | 4.77 | **<0.001** | 2.03 | **1.39 - 2.96** | 1.33 | 0.85 - 2.08 |
| **Gender** |  |  |  |  |  |  |  |  |  |
| Male | 494 | 60.39 | 1042 | 71.03 |  | 1 |  | 1 |  |
| Female | 324 | 39.61 | 425 | 28.97 | **<0.001** | 1.61 | **1.35 - 1.92** | 1.49 | **1.23 - 1.80** |
| **Ethnicity** |  |  |  |  |  |  |  |  |  |
| Others | 94 | 11.49 | 248 | 16.91 |  | 1 |  | 1 |  |
| Chinese | 631 | 77.14 | 922 | 62.85 |  | 1.81 | **1.39 – 2.34** | 1.70 | **1.31 - 2.22** |
| Malay | 42 | 5.13 | 74 | 5.04 |  | 1.50 | 0.96 – 2.34 | 1.32 | 0.84 – 2.08 |
| Indian | 51 | 6.23 | 223 | 15.2 | **<0.001** | 0.60 | **0.41 – 0.89** | 0.64 | **0.43 – 0.94** |
| **Hypertension** |  |  |  |  |  |  |  |  |  |
| No | 734 | 89.73 | 1362 | 92.84 |  | 1 |  | 1 |  |
| Yes | 84 | 10.27 | 105 | 7.16 | **0.010** | 1.48 | **1.10 – 2.00** | 1.08 | 0.73 – 1.60 |
| **Diabetes** |  |  |  |  |  |  |  |  |  |
| No | 773 | 94.50 | 1420 | 96.80 |  | 1 |  | 1 |  |
| Yes | 45 | 5.50 | 47 | 3.20 | **0.007** | 1.76 | **1.16 - 2.67** | 1.47 | 0.90 - 2.38 |
| **Hyperlipidemia** |  |  |  |  |  |  |  |  |  |
| No | 757 | 92.54 | 1378 | 93.93 |  | 1 |  | 1 |  |
| Yes | 61 | 7.46 | 89 | 6.07 | 0.198 | 1.25 | 0.89 - 1.75 | 0.78 | 0.51 – 1.20 |
| **Asthma** |  |  |  |  |  |  |  |  |  |
| No | 782 | 95.6 | 1393 | 94.96 |  | 1 |  | 1 |  |
| Yes | 36 | 4.40 | 74 | 5.04 | 0.491 | 0.87 | 0.58 - 1.3 | 0.81 | 0.53 – 1.22 |

^$^ Pearson Chi-square

^*^Adjusted odds ratio was obtained from a multivariate logistic regression being adjusted by age groups, gender, ethnicity, diabetes mellitus and hypertension.

DHF-Dengue Hemorrhagic Fever

DF- Dengue Fever

COR- Crude odds ratio

AOR- Adjusted odds ratio

CI- Confidence interval
