# Supplementary material for: Diabetes with Hypertension as Risk Factors for Adult Dengue Hemorrhagic Fever in a Predominantly Dengue Serotype 2 Epidemic: A Case Control Study
Source: PLoS Negl Trop Dis. 2012 May 1;6(5):e1641. doi: 10.1371/journal.pntd.0001641 (PMC3341340; doi:10.1371/journal.pntd.0001641)
Supplement: Table S3 — Descriptive analysis of demographics and co-morbidities variables between epidemics in year 2006 and year 2007–2008. There are significant differences in the distribution of age groups, number of adult dengue patients with diabetes, hypertension and hyperlipidemia, as well as the number of DHF cases between the group of patients admitted in year 2006 epidemic and the group admitted in year 2007–2008 epidemic. (DOC) [file pntd.0001641.s003.doc]

**Table S3.** Descriptive analysis of demographics and co-morbidities variables between epidemics in year 2006 and year 2007-2008.

| **Year** | **2006(N=475)** | | **2007-2008(N=1810)** | |  |
| --- | --- | --- | --- | --- | --- |
| **Exposures** | **N** | **%** | **N** | **%** | **P-value**^∆^ |
| **Age (Years)** |  |  |  |  |  |
| Mean (SD) | 35 | (11.7) | 37 | (13.1) | **0.014^** |
| <30 | 158 | 33.3 | 585 | 32.3 |  |
| 30-39 | 173 | 36.4 | 537 | 29.7 |  |
| 40-49 | 97 | 20.4 | 365 | 20.2 |  |
| 50-59 | 31 | 6.5 | 208 | 11.5 |  |
| ≥ 60 | 16 | 3.4 | 115 | 6.4 | **<0.001** |
| **Gender** |  |  |  |  |  |
| Female | 141 | 29.7 | 608 | 33.6 |  |
| Male | 334 | 70.3 | 1202 | 66.4 | 0.106 |
| **Ethnicity** |  |  |  |  |  |
| Chinese | 320 | 67.4 | 1233 | 68.1 |  |
| Malay | 16 | 3.4 | 100 | 5.5 |  |
| Indian | 64 | 13.5 | 210 | 11.6 |  |
| Others | 75 | 15.8 | 267 | 14.8 | 0.183 |
| **Co-morbidities** |  |  |  |  |  |
| Hypertension | 20 | 4.2 | 169 | 9.3 | **<0.001** |
| Diabetes Mellitus | 9 | 1.9 | 83 | 4.6 | **0.008** |
| Hyperlipidemia | 13 | 2.7 | 137 | 7.6 | **<0.001** |
| Asthma | 19 | 4.0 | 91 | 5.0 | 0.352 |
| **Classification** |  |  |  |  |  |
| DF | 326 | 68.6 | 1141 | 63.0 |  |
| DHF | 149 | 31.4 | 669 | 37.0 | **0.024** |

^∆^ Person’s Chi-square, unless otherwise annotated

^ Mann-Whitney U test

DHF-Dengue Hemorrhagic Fever

DF- Dengue Fever
